# ADAM10 expression in gastric adenocarcinoma: Results of a curative gastrectomy cohort

**DOI:** 10.12669/pjms.37.2.3613

**Published:** 2021

**Authors:** Huseyin Alakus, Mustafa Kaya, Hatice Ozer, Hatice Reyhan Egilmez, Kursat Karadayi

**Affiliations:** 1 Huseyin Alakus, MD. Department of Surgical Oncology, Adiyaman University Faculty of Medicine, Adiyaman, Turkey; 2 Mustafa Kaya, MD. Department of Surgical Oncology, Dr. Ersin Aslan Education & Research Hospital, Gaziantep, Turkey; 3 Hatice Ozer, MD. Department of Pathology, Cumhuriyet University Faculty of Medicine, Sivas, Turkey; 4 Hatice Reyhan Egilmez, PhD. Department of Pathology, Cumhuriyet University Faculty of Medicine, Sivas, Turkey; 5 Kursat Karadayi, PhD. Department of Surgical Oncology, Cumhuriyet University Faculty of Medicine, Sivas, Turkey

**Keywords:** ADAM10 Protein, Neoplasms, rhoA GTP-Binding Protein, Stomach Neoplasms

## Abstract

**Objective:**

Gastric cancer is among the most common human cancers with high mortality rates. ADAM10, a member of the ADAM (a disintegrin and metalloproteinase) family has also been found to be associated with gastric carcinoma and has been suggested as a potential therapeutic target. Here, we investigated the association of ADAM10 expression with prognosis in gastric adenocarcinoma patients that underwent gastric resection with D2 lymph node dissection.

**Methods:**

Total 86 consecutive patients that underwent resection for gastric adenocarcinoma were included. Immunohistochemical ADAM10 expression and its association with clinicopathological parameters were analyzed. Univariate and multivariate analyses and survival analyses were performed using SPSS ver.22.

**Results:**

High grade tumors, advanced stage tumors and diffuse type tumors showed significantly worse prognosis. A statistically significant association between ADAM10 expression and overall survival (OS) was observed in the univariate analysis, however, this association did not maintain its significance in the multivariate analysis. No statistically significant association was found ADAM-10 expression and clinicopathological parameters.

**Conclusion:**

Immunohistochemical ADAM10 expression may be used as a prognostic marker in gastric adenocarcinoma, however, introduction of a standardized immunohistochemical scoring system seems to be necessary for evaluation of ADAM10 staining.

## INTRODUCTION

Gastric cancer is the fourth most common cancer and the second leading cause of cancer-related death.^1^,^2^ Currently, surgical resection and lymph node dissection is the main treatment option for gastric cancer. Surgery also allows the staging and therefore, prognosis prediction.^3^ Improvements in overall survival (OS) rates have been relatively minor despite major developments in cancer diagnosis and treatment in the last decades, hence studies focus on biological characteristics of cancer.^4^

ADAM10 is a member of the ADAM (a disintegrin and metalloproteinase) family that is involved in cancer progression and inflammatory diseases.^5^ Other members of ADAM family are e-cadherin, epidermal growth factor (EGF), receptor tyrosine kinase 2 (ERBB2) and inflammatory cytokines.^5^ Based on the evidence showing that EGF family plays a major role in progression, invasion and metastasis of gastric cancer, the potential role of ADAM10 as a therapeutic target in cancer and inflammatory diseases has recently gained attention.^5^,^6^ EphA8, an oncogene, has been shown to induce tumor cell proliferation and migration by increasing ADAM10 expression in gastric cancer cells.^7^ Ge et al have reported that expression of microRNA-320a is inversely correlated with mRNA levels of ADAM10 and that upregulation of microRNA-320a decreases ADAM10 expression, and therefore, cell proliferation, and increases sensitivity to cisplatin in gastric cancer cells. The authors have suggested that potential therapeutic strategies for gastric carcinoma may be based on the miR-320a/ADAM10 axis.^8^

The aim of this study was to investigate the association of ADAM10 expression with prognosis and histopathological prognostic markers in patients with gastric adenocarcinoma that underwent gastric resection with D2 lymph node dissection.

## METHODS

The study protocol was approved by institutional ethics committee.(Ref. 10/02 Oct. 26, 2015.) Between February 2008 and February 2017, 144 patients that underwent gastric resection due to gastric cancer in our center were evaluated for eligibility to participate in this study. The eligibility criteria were as follows: (1) not receiving neoadjuvant chemotherapy, (2) no history of gastrectomy or other malignancies, (3) not having stage 4 gastric cancer, (4) pathologically negative surgical margins, (5) pathological diagnosis of adenocarcinoma, and (6) no surgical mortality.

The data of 86 patients who met the eligibility criteria were retrospectively analyzed. All operations were performed by an experienced surgical oncologist. Total gastrectomy was performed in proximal gastric cancer cases and distal subtotal gastrectomy in patients with distal gastric cancer. In local invasive cancers without distant metastasis, the resection of the peripheral organ (spleen, pancreas, transverse colon, etc.) was included in gastrectomy.

### Clinicopathological information and Follow-up

Clinicopathological information was retrieved from hospital records. Age, gender, type of gastrectomy, pathologic TNM stage, tumor type per Lauren classification, tumor grade and the presence of lymphovascular invasion were noted.

All patients underwent curative gastrectomy and D2 lymph node dissection and were followed up until October 2019 per the following protocol: 1) Clinical follow-up in every 3-6 months in post-surgical 2 years, 2) Clinical follow-up in every 6-12 months in post-surgical 2-5 years, and 3) Annual clinical follow-up after the fifth post-surgical year. Stage 2 and 3 cases were evaluated by computed tomography (CT) and/or positron emission tomography (PET)-CT in every 6-12 months in the first 3 years and then annually until five years.

### Immunohistochemical evaluation of ADAM10

3 μm thick sections were prepared from formalin-fixed and paraffin-embedded tissues and sections were deparaffinized and dehydrated using routine protocols. Immunohistochemical study was performed by using rabbit ADAM10 (*THERMO FISHER*; Rabbit Polyclonal, 1/100 dilution) antibodies.

**Fig.1:**
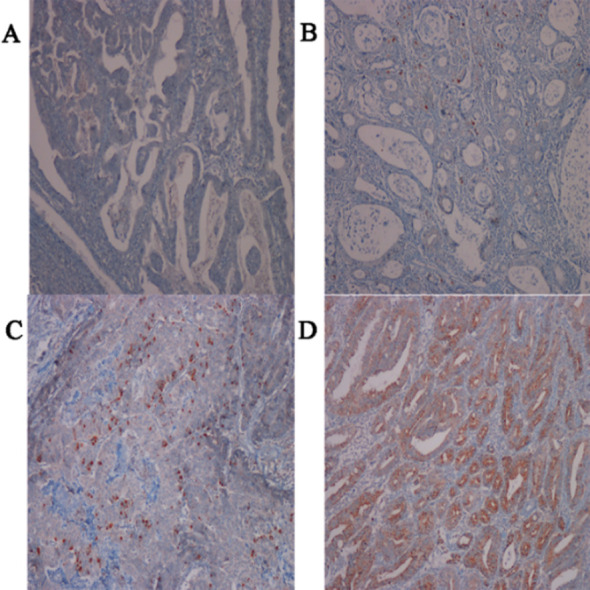
Immunohistochemical scoring of ADAM10 staining. A) 0: no staining, B) 1: 1-25% positivity, C) 2: 26-50% positivity, and D) 3: > % 50 positivity. (Immunohistochemistry, original magnification x200, x100, x200 and x100).

The percentage (distribution) of the staining within the tumor cells were scored between 0 and 3: 0: No staining; 1:1-25% (focal); 2: 26-50% (moderate); 3: >50% (diffuse) (Fig.1). The cases with scores 0 and 1 (low-expression) were compared to the cases with scores 2 and 3 (high-expression).

### Statistical analysis

Statistical analyses were performed using SPSS ver.22 (*Chicago, IL, USA*). Fisher’s exact test and/or chi-square test were used to compare categorical variables. The association between the clinicopathological variables and overall survival (OS) were analyzed by one-way ANOVA and Cox regression tests. OS was calculated using Kaplan-Meier analysis. P <0.05 was considered statistically significant.

## RESULTS

The mean age of patients was 65.85±10.4 (range 38-87 years) and the mean follow-up time was 30.69±26.3 months (range: 2-115 months). Clinicopathological characteristics are given in Table-I. No statistically significant difference was found between low and high ADAM10 expression patient groups regarding age, gender, type of gastrectomy, TNM stage, tumor type, tumor grade and/or the presence of LVI (P>0.05) .

**Table-I:** Clinicopathological features of the patients.

*Variable*	*ADAM10 expression*		*P value*

	*Negative n=61*	*Positive n=25*	
***Age (year)***			
≤65	21	9	0.889
>65	40	16	
***Gender***			
Female	11	5	0.474
Male	50	20	
***Gastrectomy***			
Total	40	14	0.404
Distal	21	11	
***pT***			
T1	7	1	
T2	7	2	1.626
T3	20	10	
T4	27	12	
***pN***			
N0	17	4	
N1	10	4	2,259
N2	12	4	
N3	22	13	
***TNM stage***			
Stage 1	11	1	
Stage 2	16	8	2.922
Stage 3	34	16	
***Lauren’s classification***			
Intestinal type	39	15	0.732
Diffuse type	22	10	
***Grade***			
Low	15	7	
Intermediate	22	7	0.517
High	24	11	
***Lymphovascular invasion***			
Absent	25	9	0.668
Present	36	16	

**Fig.2:**
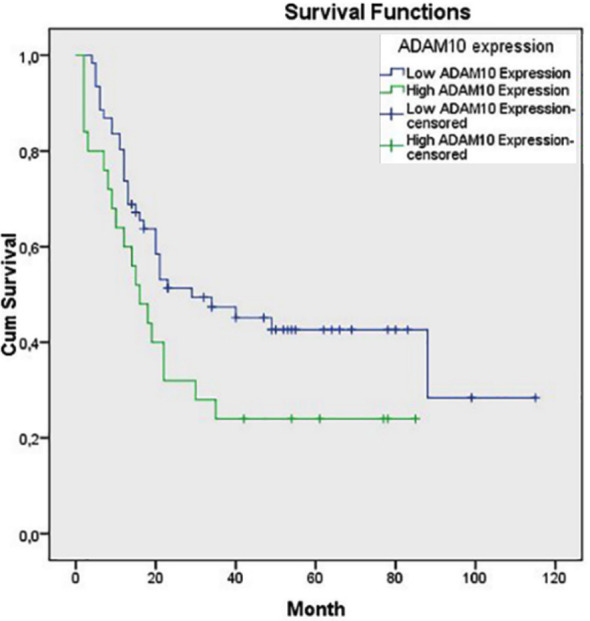
Survival comparison (Kaplan–Meier curves) of ADAM10-negative and ADAM10-positive gastric carcinomas (p=0.047).

There was significant association between OS and ADAM10 expression in Kaplan-Meier analysis (p=0.047) (Fig.2). A statistically significant association between ADAM10 expression and OS was observed in the univariate analysis (Low ADAM10 expression vs. high ADAM10 expression, p=0.008), however, this association did not maintain its significance in the multivariate analysis (Table-II).

**Table-II:** Univariate and multivariate analysis results of variables including ADAM10 expression affecting cumulative survival in patients undergoing curative surgery for gastric cancer.

*Variables*	*Univariate*			*Multivariate*	

	*n*	*P value*	*B*	*%95 CI*	*P value*
***Age (year)***					
≤65	30	0.291	1.00	0.382-1.373	0.323
>65	56		-0.322		
***Gender***					
Female	16	0.405	1.00	0.256-1.344	0.207
Male	70		-0.533		
***Gastrectomy***					
Total	54	0.684	1.00	0.618-2.062	0.692
Distal	32		0.122		
***pT***					
T1	8	0.591	1.00		0.849
T2	9		-0.189	0.100-6.848	
T3	30		0.367	0.264-7.882	
T4	39		-0.098	0.420-1.957	
***pN***					
N0	21		1.00		0.679
N1	14	0.165	0.125	0.159-8.093	
N2	16		0.791	0.372-13.087	
N3	35		0.088	0.471-2.534	
***TNM stage***					
Stage 1	12		1.00		0.674
Stage 2	24	0.041	-0.865	0.021-8.596	
Stage 3	50		-0.802	0.069-2.894	
***Tumor type (Lauren classification)***					
Intestinal type	53	0.680	1.00	0.000-1.265	0,857
Diffuse type	33		-10.313		
***Grade***					
Low	21	0.630	1.00	0.000-7.130	0.339
intermediate	28		9.836	0.000-3.779	
Severe	37		9.202		
***Lymphovascular invasion***					
No	32	0.804	1.00	0.379-1.500	0.421
Yes	54		-0.282		
***ADAM10 expression***					
Negative	61	0.008	1.00	0.312-1.082	0.087
Positive	25	-0.543			

**Fig.3:**
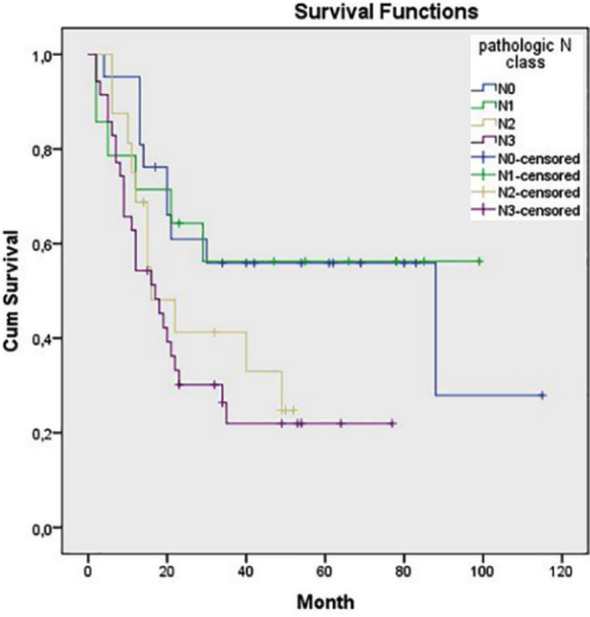
Survival comparison (Kaplan–Meier curves) of the patients according to Pathologic N stage (p=0.033).

**Fig.4:**
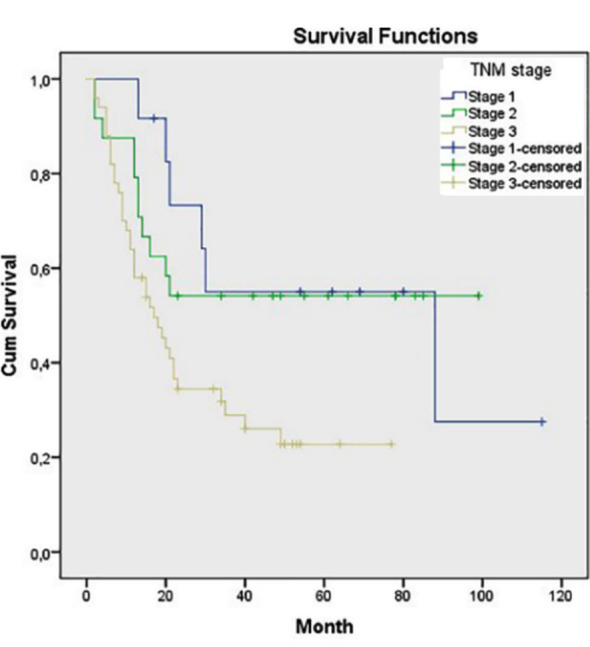
Survival comparison (Kaplan–Meier curves) of the patients according to Pathologic TNM stage (p=0.020).

**Fig.5:**
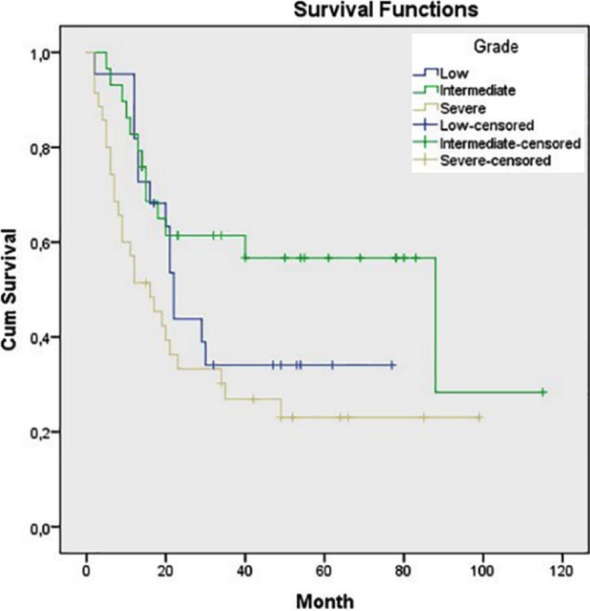
Survival comparison (Kaplan–Meier curves) of the patients according to tumor grade (p=0.027).

**Fig.6:**
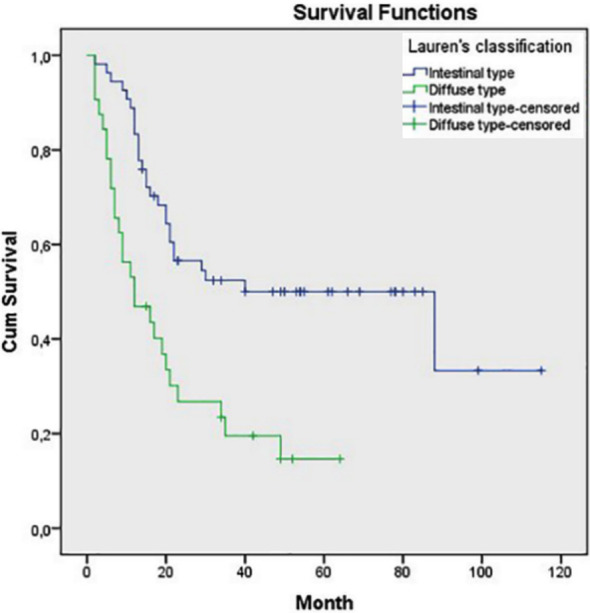
Survival comparison (Kaplan–Meier curves) of the patients according to tumor type (intestinal type vs. diffuse type) (p<0.001).

Statistically significant associations were also found between OS and pN class, pTNM stage, tumor grade and tumor type as diffuse type had worse prognosis (p=0.033, p=0.020, p=0.027 and p<0.001, respectively) (Fig.3-6). Tumor grade was also found to be a significant factor in univariate analysis (p=0.041) but not in multivariate analysis (p>0.05).

## DISCUSSION

ADAM10 overexpression has been detected in various cancer types.^9^-^14^ Knösel et al have found that immunohistochemical ADAM10 expression is correlated with advanced stage in colorectal cancer patients.^15^ Interestingly, Gavert et al have reported that ADAM10 mediates the formation of liver metastases in mice with colorectal carcinoma.^16^ Yoshimura et al have found that ADAM10 and ADAM17 expressions are increased in Helicobacter pylori gastritis and the authors have concluded that ADAM10 may be involved in gastric carcinogenesis in Helicobacter pylori infected patients.^17^ Similar to our study, Wang et al have studied the ADAM10 expression in curative gastrectomy samples and they have shown that ADAM10 overexpression is an important prognostic marker in gastric cancer patients as it is significantly associated with age, tumor size, tumor location, depth of invasion, lymphovascular invasion and TNM stage.^18^ However, we observed a significant association between ADAM10 expression and overall survival only in the univariate analysis and we did not find any significant association between ADAM10 expression and other clinicopathological parameters.

Lymph node metastasis is one of the most important prognostic factors in gastric cancer and advanced TNM stage is also an unfavorable prognostic factor in patients with gastric carcinoma.^19^-^23^ As expected, we also found that pN and advanced pTNM stage had a significant negative impact on overall survival.

Other parameters that we demonstrated to be associated with overall survival were tumor type and tumor grade. The Lauren classification demonstrates numerous differences in etiology, epidemiology and pathology of gastric cancer and diffuse type gastric carcinomas often show a poor prognosis.^24^-^27^ We observed that diffuse type gastric cancers have a poorer prognosis with shorter overall survival compared to intestinal type gastric cancer, consistent with the literature. Regarding tumor grade, we had conflicting results as we found a significant association between tumor grade and prognosis in Kaplan-Meier analysis and no significant association between tumor grade and survival in univariate and multivariate analyses. Similarly, Hu et al have not observed a statistically significant association between tumor grade and survival in uni- and multivariate analyses.

### Limitation of the study

Our study group was consisted of only 86 patients and ADAM10 showed cytoplasmic staining. There are studies in the literature reporting cytoplasmic staining.^7^,^12^,^18^ Although we did not compare the significance of cytoplasmic staining with the significance of membranous or nuclear staining in the present study, we think that comparison of the staining patterns may also provide useful information. Finally, there is no consensus on how to score ADAM10 expressions in the literature.^12^,^18^,^28^ Different scoring systems have been adapted for evaluation of ADAM10 expression. ADAM10 expression has been scored between 0-3 based on the staining percentage/tumor cell ratio in a study by Wang et al..^18^ Ko et al grouped the ADAM10 expression results as <10% (negative), 10-50% (weak staining) and >50% (strong staining).^28^ We described ≥26% as positive ADAM10 expression. Therefore, we think that small number of the patients and differences in immunohistochemical scoring may have affected our results.

## CONCLUSION

In this study, we evaluated the association between the clinicopathological prognostic parameters and ADAM10 expression and our results show that ADAM10 expression may be used as a prognostic marker in gastric adenocancer.

### Author’s Contributions:

**HA:** Data collection, conception, preparing the manuscript and is responsible for integrity of the study.

**MK:** Data collection and writing the article.

**HO:** Interpretation of data.

**HRE:** Interpretation of data.

**KK:** Revision for important intellectual content, supervision.
